# Massive pneumoretroperitoneum arising from emphysematous cholecystitis: a case report and the literature review

**DOI:** 10.1186/s12876-015-0345-8

**Published:** 2015-09-08

**Authors:** Yasumichi Yagi, Shozo Sasaki, Itsuro Terada, Akemi Yoshikawa, Wataru Fukushima, Hirohisa Kitagawa, Takashi Fujimura, Ryohei Izumi, Katsuhiko Saito

**Affiliations:** 1 Department of Surgery, Toyama City Hospital, 2-1 Imaizumi Hokubu-machi, Toyama, 939-8511 Japan; 2 Department of Pathology, Toyama City Hospital, 2-1 Imaizumi Hokubu-machi, Toyama, 939-8511 Japan

## Abstract

**Background:**

Emphysematous cholecystitis is a severe variant of acute cholecystitis caused by anaerobic bacteria. Although intraperitoneal air as a complication has been described in association with emphysematous cholecystitis, pneumoretroperitoneum arising from emphysematous cholecystitis is extremely rare. Herein, we describe a rare case of pneumoretroperitoneum arising from emphysematous cholecystitis that was successfully treated with emergency surgery.

**Case presentation:**

An 84-year-old male was transported to the Emergency Department of our hospital for acute abdomen. Computed tomography revealed acute cholecystitis accompanied by emphysematous change. Computed tomography also revealed massive pneumoretroperitoneum complicated with pneumobilia and gas in the hepatoduodenal ligament. Clinical findings fulfilled the diagnostic criteria for systemic inflammatory response syndrome and sepsis. Emergency surgery was carried out with a diagnosis of both emphysematous cholecystitis and gastrointestinal perforation. Intraoperative findings revealed acute gangrenous cholecystitis and pneumoretroperitoneum presenting with an odor-free foamy abscess along the loose connective tissue behind the ascending colon and mesocolon. No evidence of gastrointestinal perforation was found during surgery. Therefore, cholecystectomy and lavage drainage were performed. Bacterial culture examination isolated a single species of anaerobe, *Klebsiella pneumoniae*, which was considered to be the cause of emphysematous cholecystitis, pneumobilia, and pneumoretroperitoneum.

**Conclusions:**

Emphysematous cholecystitis should be considered as a possible cause of pneumoretroperitoneum. The present case is the first report of massive pneumoretroperitoneum extending to the dorsal side of the ascending mesocolon as a complication of emphysematous cholecystitis.

## Background

Emphysematous cholecystitis (EC) is a rare, severe, potentially lethal variant of acute cholecystitis characterized clinically by gas accumulation in the wall and lumen of the gallbladder or pericholecystic tissue [1, 2]. It is caused by gas-forming organisms, especially anaerobes, such as *Escherichia coli, Aerobacter aerogens, Klebsiella spp, Clostridium spp,* and *Salmonella spp*, leading to severe complications: gangrene, perforation of the gallbladder, and pericholecystic abscess [3, 4]. The mortality rate due to EC is reportedly 25 % [2], which is 4 % higher than all types of acute cholecystitis [3].

Extra-intestinal gas, which strongly suggests perforation of the gastrointestinal tract, is a critical sign of the need for emergency surgery. Previous reports have indicated that EC leads to the development of intraperitoneal air over the progression of anaerobic infection [4, 5]. However, pneumoretroperitoneum complicated with EC is extremely rare. Herein, we describe a rare case of pneumoretroperitoneum arising from EC that was successfully treated with emergency surgery.

## Case presentation

**Fig. 1:**
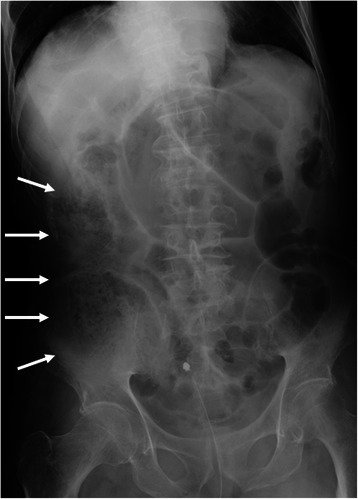
An abdominal X-ray. In the spine position, an irregular gas image was observed in the right lateral abdomen, corresponding to pneumoretroperitoneum (arrows)

**Fig. 2:**
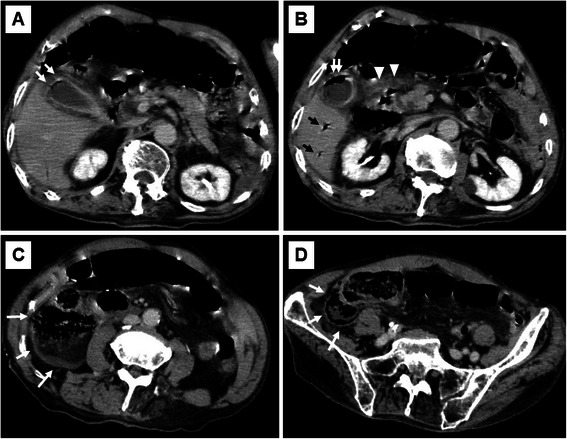
Abdominal CT findings. **a**: CT showed a thickened wall and intramural air of the gallbladder, suggesting emphysematous cholecystitis (white arrows). **b**: Pneumobilia was detected in the intrahepatic bile duct (black arrows). The gallbladder contained air within the lumen (white arrows). Subserosal free air was detected in the hepatoduodenal ligament and around the pancreas head (white arrowheads). **c**: Massive pneumoretroperitoneum was observed behind the right-sided colon (white arrows). **d**: Pneumoretroperitoneum extended to the retroperitoneal space behind the ascending mesocolon, bordered by the cecum and iliopsoas muscle (white arrows)

**Fig. 3:**
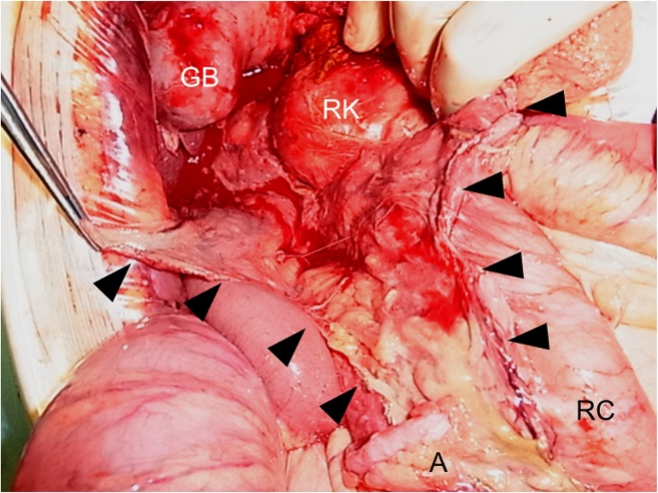
Intraoperative photograph. After mobilization of the right-sided colon, the retroperitoneal space became obvious with an odor-free foamy abscess along the loose connective tissue of the retroperitoneum (black arrowheads). The gallbladder exhibited acute gangrenous cholecystitis with a destructed wall. GB: gallbladder, RK: right kidney, A: appendix, RC: right-sided colon

**Fig. 4:**
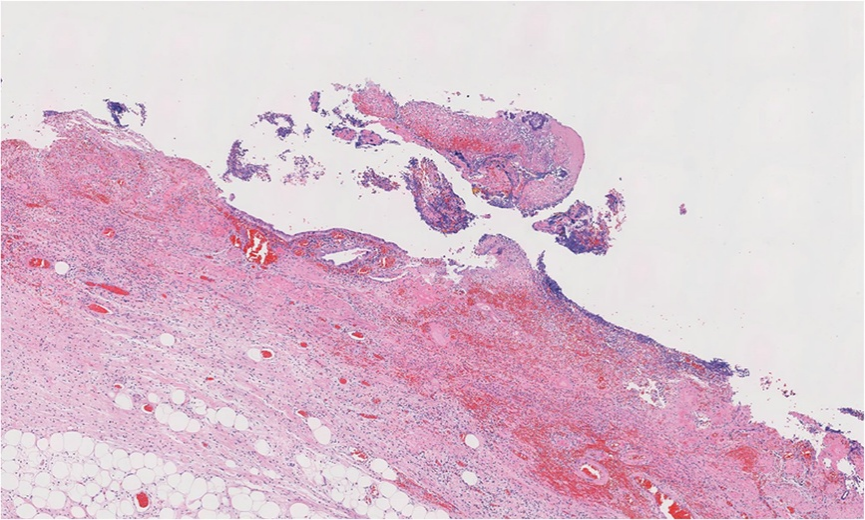
Microscopic finding of the resected specimen (H-E staining, ×100). The GB wall exhibited acute suppurative inflammation: hemorrhagic, necrotic changes with neutrophil infiltration

An 84-year-old male was transported to the Emergency Department of our hospital for abdominal pain and vomiting. He had a history of hypertension and cerebral infarction. His vital signs were as follows: pulse, 136 beats/min; blood pressure, 154/75 mmHg; saturation, 93 % in room air; and body temperature, 36.9 °C. His abdomen was not distended, but there was local tenderness with muscular defense in the right upper abdomen. Laboratory data showed a white blood cell count of 26,800/μL (normal level, <8500/μL) with 91.0 % neutrophils, hemoglobin of 15.4 g/dL (normal level, >13.5 g/dL), glycated hemoglobin A1c (HbA1c) of 6.9 % (National Glycohemoglobin Standardization Program) (normal level, <6.2 %), fasting blood sugar of 195 mg/dL (normal level, <109 mg/dL), aspartate aminotransferase of 23 IU/L (normal level, <31 IU/L), alanine aminotransferase of 12 IU/L (normal level, <40 IU/L), alkaline phosphatase of 249 IU/L (normal level, <210 IU/L), total bilirubin of 1.2 mg/dL (normal level, <1.2 mg/dL), γ-guanosine triphosphate of 17 IU/L (normal level, <73 IU/L), serum creatinine of 1.23 mg/dL (normal level, <1.1 mg/dL), and C-reactive protein of 5.93 mg/dL (normal level, <0.3 mg/dL). Urinary analysis showed 1+ glucose and 3+ bacteria. Arterial blood gas analysis showed respiratory alkalosis and decreased pCO_2_ of 25.5 mmHg (normal level, <32 mmHg). These clinical findings fulfilled the diagnostic criteria for systemic inflammatory response syndrome and sepsis. An abdominal X-ray showed irregular gas accumulation in the right lateral abdomen (Fig. 1). Computed tomography (CT) indicated stones and gas accumulation in the thickened wall and lumen of the gallbladder. CT also detected pneumobilia, subserosal gas around the hepatoduodenal ligament, and massive pneumoretroperitoneum behind the ascending mesocolon (Fig. 2). A diagnosis was difficult to make because we could not exclude the possibility of perforation of the duodenum and large intestine. Thereafter, emergency surgery was carried out with a diagnosis of both EC and gastrointestinal perforation. Intraoperative findings revealed acute cholecystitis; the gallbladder wall was destructed with severe inflammation, especially in the neck of the gallbladder. With mobilization of the right-sided colon and mesocolon, an odor-free foamy abscess was observed along the loose connective tissue of the right retroperitoneum and extended continuously to the hepatoduodenal ligament and Calot’s triangle (Fig. 3). Unexpectedly, no evidence of perforation was observed in the stomach, duodenum, or large intestine during surgery. Therefore, we performed cholecystectomy with lavage drainage. Postoperative management of the infection was performed with administration of meropenem three times of 0.5 g for 10 days. Because laboratory data fulfilled the criteria of disseminated intravascular coagulation (The revised Japanese Association for Acute Medicine) [6], recombinant human thrombomodulin was administered to improve sepsis-induced disseminated intravascular coagulation for 5 days. He was medicated with a ventilator because of aspiration pneumonia, and was weaned off ventilatory support by day 5 after surgery. Histological findings indicated acute cholecystitis with gangrenous change (Fig. 4). *Klebsiella pneumoniae* was isolated from the culture of the abscess, and it was considered the cause of EC, pneumobilia, and pneumoretroperitoneum. Eventually, he recovered and was transferred to another rehabilitation hospital.

## Discussion

Most ECs occur in patients aged 50 ~ 80 years [7]. Unlike common cholecystitis, the prevalence of EC in males is about three times that in females [8]. Approximately 50 % of them have diabetes mellitus (DM) and peripheral vascular disease [9]. DM is also well known as a predisposing factor of gas gangrene [10], not only in EC but also in emphysematous pancreatitis [11] and emphysematous pyelonephritis [12]. High glucose level yields a microenvironment which promotes growth of anaerobic bacteria, producing gas under hypoxia of the ischemic tissue in such diseases [13]. In the present case, the patient had no history of DM, but was complicated by high blood sugar level with elevation of HbA1c. Under control of the infection by antibiotics and insulin, blood sugar was decreased down to a normal level. Pneumoretroperitoneum in the present case is considered to have derived from the progression of EC in relation to the diabetic and hypoxic condition of the patient.

In the diagnosis of cholecystitis, ultrasound is indeed available and widely conducted for detection of earlier stage cholecystitis. With regard to EC, the previous reports indicated that the air in the wall and lumen of the gallbladder interfered in visualization of ultrasound image with a reverberation artifact [2]. Besides it is difficult to tell whether air was in the lumen or in the wall of the gallbladder. In the diagnosis of EC, CT is of great help for detection of air with higher sensitivity and specificity compared with other radiological modalities [13]. A final diagnosis, however, requires the demonstration of an anaerobe in bacterial culture examination. In addition to anaerobic infection, pneumoretroperitoneum can be caused by gastrointestinal perforation, such as perforation of the duodenal diverticulum [14] and large intestine [15]. Except for gastrointestinal perforation, emphysematous pancreatitis [11] and emphysematous pyelonephritis [12] are considered as differential diagnoses of pneumoretroperitoneum. In principle, the distribution of the air presents along the organ of the disease origin on CT images. As a matter of course, whenever we encounter a patient with acute abdomen, the presence of retroperitoneal air leads to the first assumption of gastrointestinal perforation. If gastrointestinal perforation is suspected, emergency surgery with open drainage would be required even for EC with pneumoretroperitoneum. Although we rarely encounter a patient with pneumoretroperitoneum arising from EC, a definite diagnosis should be made in order to make a determination on emergency surgery. In the present case, we could not exclude gastrointestinal perforation. If the diagnosis could be made as EC, percutaneous drainage guided by ultrasound or CT would be a treatment option for both gallbladder and pneumoretroperioneum. However, in the present case, retroperitoneal abscess was spread widely with sponge-like septum. Thereafter, we should take account of probability that percutaneous drainage resulted in insufficient drainage, if the retroperitoneal abscess was not localized to a solitary cavity. For elderly high-risk patients, percutaneous drainage would be a treatment option as a second best alternative [16].

Extra-luminal gas complicated with EC has been conventionally considered to derive from gallbladder perforation [17]. Whenever presented with EC, pneumoretroperitoneum might occur following gallbladder perforation with anaerobic infection. In the present case, macroscopic gallbladder perforation was not clarified in intraoperative findings. However, the resected gallbladder revealed gangrenous cholecystitis with destruction of the wall, which supported the presence of microperforation of the gallbladder or infectious spread of an anaerobe in the subserosal layer. This theory is supported by cases of pneumoperitoneum without perforation of the gallbladder in EC. A literature review identified 16 cases of EC with pneumoperitoneum. However, the finding of macroscopic perforation of the gallbladder was found in only eight cases [4]. Hence, pneumoperitoneum derived from EC may occur regardless of macroscopic gallbladder perforation. As for pneumoretroperitoneum, Orland et al. reported a case of EC complicated with retroperitoneal gas instead of gallbladder perforation [18]. Therefore, EC should be considered as a possible cause of pneumoretroperitoneum, even without perforation of the gallbladder.

Pneumoperitoneum and pneumoretroperitoneum as complications, which represent extended infections, seem to be obviously more severe condition compared with EC localized in the gallbladder. Gill et al. divided EC radiographically into three stages according to the distribution of air within the gallbladder and/or the biliary system as follows: Stage1 –air in the gallbladder lumen, Stage2 –air in the gallbladder wall, and Stage3 –air in the pericholecystic tissue [19]. Accordingly, the present case corresponded to the stage3. Some studies have indicated that the spread of gangrene does not always relate to a poorer prognosis [20]. In the present case, massive gas might have reflected on the progression of infection because the intraoperative findings showed wide-spreading gangrene of retroperitoneum and the postoperative course indicated a severe general condition. Therefore, pneumoperitoneum and pneumoretroperitoneum should be regarded as life-threatening and an extensive infectious form of EC. Further examination of EC is required and the relationship between pneumoretroperitoneum and its prognosis should be discussed in the future.

Progression of EC can cause the spread of gas to the hepatoduodenal ligament [18] or escape into the peritoneal cavity [21]. From an anatomical viewpoint, retroperitoneum, hepatoduodenal ligament, and pericholecystic tissue are connected by fascial planes. Such a connection between the pericholecystic tissue and retroperitoneum allows air to escape from the gallbladder when the gas pressure increases in weak connective tissues, and this may explain the pathway in which cholecystic air, produced due to anaerobic bacterial infection, reaches the retroperitoneum. Pneumoperitoneum arising from EC itself is very rare. Pneumoretroperitoneum associated with EC is extremely rare. Previous reports have identified only one case of pneumoretroperitoneum due to EC. However, this case presented a little subserosal gas around the hepatoduodenal ligament and pancreas head. A large amount of pneumoretroperitoneum complicated with EC has not yet been reported, except for the present case.

## Conclusion

EC should be considered as a cause of pneumoretroperitoneum. Whenever we encounter pneumoretroperitoneum concurrently with EC, it should be assumed that retroperitoneal gas might have derived from gas-producing anaerobic infection of the gallbladder extending to retroperitoneum. The present case is the first report of massive pneumoretroperitoneum extending to the dorsal side of the ascending mesocolon as a complication of EC.

## Consent

Written informed consent was obtained from the patient for publication of this case report and any accompanying images. A copy of the written consent is available for review by the Editor-in-Chief of this journal.

## Abbreviations

EC: Emphysematous cholecystitis
CT: Computed tomograph
HbA1c: Glycated hemoglobin A1c
DM: Diabetes mellitus
